# A study of health effects of long-distance ocean voyages on seamen using a data classification approach

**DOI:** 10.1186/1472-6947-10-13

**Published:** 2010-03-10

**Authors:** Yunmei Lu, Yanhong Gao, Zhongbo Cao, Juan Cui, Zhennan Dong, Yaping Tian, Ying Xu

**Affiliations:** 1 College of Computer Science and Technology, Jilin University, Changchun, Jilin, 130012, China; 2 Department of Clinical Biochemistry, Chinese PLA General Hospital, Beijing, 100853, China; 3 Computational Systems Biology Laboratory, Department of Biochemistry and Molecular Biology, University of Georgia, Athens, GA, 30602, USA; 4 Institute of Bioinformatics, University of Georgia, Athens, GA, 30602, USA

## Abstract

**Background:**

Long-distance ocean voyages may have substantial impacts on seamen's health, possibly causing malnutrition and other illness. Measures can possibly be taken to prevent such problems from happening through preparing special diet and making special precautions prior or during the sailing if a detailed understanding can be gained about what specific health effects such voyages may have on the seamen.

**Methods:**

We present a computational study on 200 seamen using 41 chemistry indicators measured on their blood samples collected before and after the sailing. Our computational study is done using a data classification approach with a support vector machine-based classifier in conjunction with feature selections using a recursive feature elimination procedure.

**Results:**

Our analysis results suggest that among the 41 blood chemistry measures, nine are most likely to be affected during the sailing, which provide important clues about the specific effects of ocean voyage on seamen's health.

**Conclusions:**

The identification of the nine blood chemistry measures provides important clues about the effects of long-distance voyage on seamen's health. These findings will prove to be useful to guide in improving the living and working environment, as well as food preparation on ships.

## Background

Ocean-going seamen are living on a ship with a confined environment for a long period of time. Such a special environment, often not most human friendly, may cause various changes in the human body for people who work and live there for an extended period of time [1]. Seamen may experience subtle changes in physiological [2-5] and psychological functions [6,7] in their bodies. Many studies have been conducted on different aspects of maritime health issues. It has been previously reported that ocean voyages could affect the human immune system and result in various illness [8,9]. Specifically, it is found that the risk of ischemic heart disease (IHD) lethality on board is much higher than that on land, based on an analysis of data of 124 seamen who died suddenly of myocardial infarction [10]. The diet and the lack of physical exercises, while living on ship, are believed to be the top two contributing factors to IHD [11]. Besides, cardiovascular disease is another serious maritime health problem [9]. We expect that these illnesses and/or changes in physiological conditions will be reflected by changes in some blood chemistry measures of the seamen. In this paper, we focus on the identification of the most significantly changed chemistry measures in the seamen blood samples that we have collected before and after their ocean voyage.

Statistical methods have been often used to analyze the health effects of long-distance ocean voyages on seamen [12,13]; however, simple statistical methods are often found to be inadequate for dealing with complex relationships among physiological and psychological functions in seamen bodies under study. In this paper, we present a computational study of this type of problem using an approach different from the traditional statistical methods. We consider the problem of identifying the health effects of ocean voyages on seamen as a classification problem, i.e., to classify blood chemistry measures that are consistently affected or not by ocean voyage, and apply a supervised machine learning method, specifically support vector machines (SVM), to solve the classification problem. Support vector machines have been widely used for classification problems and found to be particularly effective for discovering informative feature patterns for small data sets [14].

The identification of discriminant features (e.g., chemistry measures of blood) among pre-defined classes of objects is of fundamental and practical interest. By identifying relationships linking specific features (e.g., certain blood chemistry measures) and feature values with certain classes of objects such as diseases, one can possibly derive new insights about the disease and its development.

For our problem, we have used a feature selection method in conjunction with the SVM-based classifier, called recursive feature elimination (RFE), to find blood chemistry measures that show consistent and substantial changes caused by ocean voyage, which takes into consideration of mutual information between features in the feature selection process. This procedure has proved to work better than other correlation-based methods [14] for solving similar problems. We anticipate that our findings, in terms of identified blood chemistry measures with the most substantial and consistent changes in seamen bodies due to ocean voyage, will provide useful guiding information for food preparation and intake for seamen during ocean voyages and for better designing of a healthier living and working environment on ship.

## Methods

Given is a collection of M seamen blood chemistry data **x**_1_, **x**_2_,... **x**_*k*_, ... **x**_*m*_, with each x_k _representing a vector  of 41 blood chemistry measures for each seaman, either before or after the ocean voyage. Our goal is to identify a subset of the 41 measures that show substantial and consistent changes caused by the ocean voyage. We formulate this problem as follows. For each x_*k *_(a seaman) in {**x**_1_, **x**_2_,... **x**_*k*_, ... **x**_*m*_}, we assign a label y_*k *_= +1 or -1 to indicate if this data is measured before or after an ocean voyage. Our classification goal is to find a discriminant function F(x) on the set of 41-dimentional vectors to best separate the samples labeled with +1 from the ones labeled with -1. A by-product in solving this classification problem is the identification of a subset of the 41 blood measures that can best distinguish between the subsets of samples with different labels, the ultimate goal of this study. Specifically, we intend to find a linear discriminant function as follows:(1)

that can best separate the two subsets with different y labels of the given seamen blood samples, where **ω **is a weight vector and b is a bias value, to be determined through finding the optimal discriminant function. For this problem, we have chosen to use an SVM approach coupled with an RFE procedure, to find the optimal discriminant function F(x), as well as a subset of blood measures that show substantial and consistent changes between the two subsets.

### Support vector machine

SVMs are a class of machine learning techniques [15] widely used for solving classification problems like our problem. An SVM-based classification algorithm constructs a separating hyperplane between two classes of data samples like the ones mentioned above with different labels in the input space. The separating hyperplane is determined by

a) mapping the input space into a higher dimensional feature space through a kernel function, and

b) constructing in this feature space two maximal margin hyperplanes [16] to separate the mapped data samples in the higher dimensional space.

**Figure 1:**
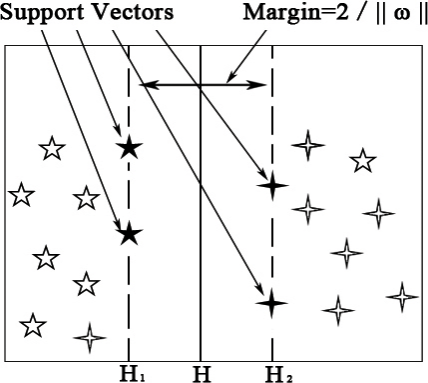
**An example of a dataset in two-dimensional space**. A dataset with two types of samples, represented by pentagram and four pointed star, respectively, where H is a separating hyperplane, and H_1_, H_2 _are two supporting hyperplanes.

The goal in training an SVM is to find a separating hyperplane along with two parallel supporting hyperplanes, one on each side of the separating hyperplane, which give the margins of the data samples to the separating hyperplane as large as possible (see Figure 1). As shown in Figure 1, the margin is equal to 2/||*ω*||; therefore, finding the hyperplanes that separate the data samples with different labels that have the maximal margin is equivalent to solving the following constrained optimization problem:(2)

with the constraints,(3)

where C>0 is a penalty parameter and ξ >0 is a slack variable. This problem is often represented in its dual form as follows:(4)

with constraints,(5)

where α's are the Lagrange multipliers. This formulation, where data vectors are present only in dot products in the feature space, makes the execution of the training algorithm simpler [15]. Specifically, the classification problem can be rewritten as,(6)

where *K *(**x **_*h*_, **x**_*k*_) is the kernel function that maps a data vector in the input space to a higher dimensional feature space, and SVs are all possible support vectors on the parallel hyperplanes as mentioned above. For further details of support vector machines, we refer the reader to [16].

### Recursive feature selection

Recursive feature elimination (RFE) is a feature-selection procedure, used in conjunction with the training of an SVM-based classifier [14], and has been applied in several microarray gene-expression studies [14,17] for feature selection and in industrial process analyses to determine the most essential process variables [18]. During the training of an SVM-based classifier, RFE is used to eliminate features that are "insignificant" to the performance of the classifier (in our case, blood measures showing no consistent and substantial differences between the before- and the after-sailing blood samples), which typically consists of three steps [14]:

-- train a classifier using an SVM;

-- compute the ranking for all features, based on some pre-defined criteria, and

-- remove the feature with the lowest ranking.

The following function *DJ() *calculates the effect on the objective function (6) by removing a specific feature, which is used in our feature selection,(7)

where H is the matrix with elements **y**_*h*_**y**_*k *_*K*(**x**_*h*_, **x**_*k*_), **H**(-*i*) is H with the i^th ^feature removed, and K is a kernel function that measures the similarity between **x**_*h *_and **x**_*k*_We used a Gaussian radial basis kernel function K (**x**_*h*_, **x**_*k*_) = exp(-γ ||**x**_*h*_, **x**_*k*_||^2^)in this study.

In this procedure, features are removed one at a time, and then the SVM will be retrained to update the new ranking of features. It should be noted that the top ranked features are not necessarily the ones that are individually most relevant to the classification performance since the relevance of features are evaluated in the context of other features, i.e., mutual information among features in terms of their collective discerning power are considered.

### Data set

Seamen's Blood Chemistry Data

200 seamen are involved in this study, who are healthy Han Chinese males with age from 19 to 38 (mean 25.2 ± 4.9). Before and after a 3-month voyage, 5 milliliters of venous blood was collected from each of the 200 seamen after fasting for 12 hours, and then has been centrifuged for 5 minutes. Serum was collected and analyzed using a HITACHI 7600 modular full automatic biochemical analyzer, for 41 chemistry measures (as listed in Table 1). (The seamen blood chemistry data is available upon request). By removing those with any missing information, a total of 170 pre-sailing and 170 post-sailing samples have been complied with completed blood chemistry measures. In our computational study, each seaman sample is represented as a feature vector consisting of 41 blood chemistry measures.

**Table 1:** 41 Blood chemistry measures used in this study

Index	1	2	3	4	5	6	7	8	9	10	11
Feature	TP	ALB	ALT	AST	TB	GLU	UN	Cr	UA	ALP	GGT

Index	12	13	14	15	16	17	18	19	20	21	22

Feature	CK	LDH	HDL	LDL	Ca	PHOS	Mg	CHOL TG	TCO_2_	UIBC	

Index	23	24	25	26	27	28	29	30	31	32	33

Feature	Fe	APOA1	APOB	CK-MB	APOA2	APOC2	APOC3	APOE	LP(a)	MAO	PLIP

Index	34	35	36	37	38	39	40	41			

Feature	FRUC	DB	TBA	ADA	Na	K	Cl	TIBC			

### Generation of training and test Datasets

Our goal is to identify a subset of blood chemistry measures among the 41 measures that show consistent and significant changes across the seamen's blood samples collected before and after sailing. We first split the whole dataset randomly into two subsets, one for training and another for testing. A training set is used to select features (blood chemistry measures) and find the right weights of the features so an optimal separating hyperplane between the two labeled subsets can be derived, while the test set is used to evaluate the effectiveness of the trained SVM mostly for its generality, where the evaluation criterion on the trained SVM is the sign function in Eq.(6).

We first mix all the blood chemistry data, both pre- and post-sailing data, into one set while keeping the "pre-" and "post-sailing" label (-/+) for each vector, and then we separate this dataset into a training and a test set, by randomly putting sample data into the two subsets. One key to establish a good training set is that it should capture all the varieties existing in our seamen samples. In order to accomplish this, we have used multiple training sets and the associated test sets to assess each trained classifier, and used a combined classifier based on all the trained individual classifiers (based on specific training set) using a majority-rule vote at the end. In order to obtain good training sets through random partition of the original dataset, we considered seven different ratios between the numbers of samples in the training and the test set, ranging from 1:1, 1.5:1, 2:1, 2.5:1, 3:1, 3.5:1, to 4:1, respectively. In total, we generated 300 pairs of training sets and associated test sets, and trained a classifier for each of the 300 training sets. Note that samples with the same or similar pre- and post- observed values but different labels are considered as noise affecting the performance. We checked our dataset to ensure that no two vectors have conflicting labels. Patients in this study all signed the Informed Consent Form; and this study has been approved by the Medical Ethics Committee in Chinese PLA General Hospital, China.

## Results and Discussion

By running the SVM-RFE procedure outlined in the above section using the 300 training sets, we obtained 300 ranked lists of features in terms of their level of contribution to our classification problem as defined in Eq.(7). Each list includes a subset of top features that make the classifier have the best classification performance, which is measured using the overall prediction accuracy P = (TP+TN)/M, with TP, TN being the numbers of true positive and true negative, respectively, and M the total number of seamen in the test set. For example, in one of the 300 results, we obtained the best overall accuracy 98.3% when 10 features are selected and used as the most useful subset of features. The 10 features are ranked as shown in Table 2. We do the same on each of the 300 training sets, and got 300 subsets of features, each of which makes its respective classifier have the best prediction performance.

**Table 2:** A ranking list by one classifier trained on one specific training dataset

Ranking order	Feature (index)
1	MAO(32)

2	GLU(6)

3	PHOS(17)

4	TCO_2_(21)

5	Ca(16)

6	FRUC(34)

7	LDH(13)

8	K(39)

9	CK-MB(26)

10	UN(7)

### Majority-rule for selecting important features

As discussed above, different ways of partitioning the original dataset into training and test sets may lead to (somewhat) different performance by the trained classifier. We have generated 300 pairs of training and test sets, and trained an SVM-based classifier for each training set. To decide the ultimate subset of features to use for getting the best classifier, we have used a majority-rule voting strategy. The premise of this strategy is that the intrinsically important features will always be chosen by the best trained classifier, which should be independent of the specific sampling. The majority-rule voting process is described as follows: for each of the 41 features, we count the number of times when this feature is among the remained features for the ith training set, i = 1, 2, ..., 300. For example, feature MAO (32) is present in all the 300 subsets, so its count is 300, while the count of feature PHOS (17) is 286. After we get the count for each of the 41 features, we re-rank all the selected features based on this count, as shown in "Count" column of Table 3.

By using the majority-rule, we found that 12 such features are chosen by at least 50% (majority) of the 300 trained classifiers, with their names along with their frequencies of being used across different trained classifiers being listed in Table 3. Then we examined different combinations of the 12 selected features to calculate the average prediction accuracy of the 300 classifiers, and the classification results of the best combinations are given in Table 3. Among these, the highest accuracy is 95.74% when all the 300 classifiers are trained on 9 features {MAO, PHOS, CK-MB, Ca, LDH, FRUC, K, Na, ALB}. These 9 features constitute the smallest subset of features that make the classifiers have the best prediction performance, so we select them as our selected subset of features [19,20].

**Table 3:** Most commonly used features and associated prediction accuracy

Ranking Order	Feature (Index)	Count (percentage %)	Features used in all classifiers	Mean Accuracy(%)
1	32(MAO)	300(100.0)	32	87.37

2	17(PHOS)	286(95.33)	32+17	90.29

3	26(CK-MB)	240(80.0)	32+17+26	93.7

4	16(Ca)	236(78.67)	32+17+26+16	93.44

5	13(LDH)	233(77.67)	32+17+26+16+13	94.12

6	34(FRUC)	226(75.33)	32+17+26+16+13+34	94.94

7	39(K)	210(70.0)	32+17+26+16+13+34+39	95.28

8	38(Na)	203(67.67)	32+17+26+16+13+34+39+38	95.49

9	2(ALB)	184(61.33)	**32+17+26+16+13+34+39+38+2**	**95.74**

10	6(GLU)	168(56.0)	32+17+26+16+13+34+39+38+2+6	90.5

11	36(TBA)	162(54.0)	32+17+26+16+13+34+39+38+2+6+36	90.64

12	5(TB)	150(50.0)	32+17+26+16+13+34+39+38+2+6+36+5	90.56

### Comparison with t-test-based feature selection

As a comparison, we have also used a paired t-test, a popular statistical method, to evaluate each feature independently in the seamen data to identify the ones that show substantial changes before and after the sailing. For each of the 41 blood chemistry measures, we have a null hypothesis that the measure has no substantial change before and after the sailing at a specified significance level α, say, α = 0.05. We use p(x) to represent the probability, under the null hypothesis, of observing x at this significance level. If p(x) >α, the test fails to reject the null hypothesis, meaning that there is no significant change before and after the sailing; if p(x) <α, the null hypothesis will be rejected, indicating that the corresponding feature has significant changes before and after the sailing. We have calculated the p values for each of the 41 features; Table 4 shows the identified significant features using α = 0.05.

**Table 4:** Blood measures chosen by the t-test with p-value < = 0.05

**No**.	Feature(Index)	**Before**()	**After**()	p-value
1	32(MAO)	0.85 ± 0.21	0.36 ± 0.18	6.26E-60

2	17(PHOS)	1.17 ± 0.17	1.39 ± 0.16	2.17E-39

3	13(LDH)	186.02 ± 37.99	158.20 ± 28.07	8.56E-18

4	10(ALP)	81.64 ± 20.44	74.34 ± 18.46	9.78E-18

5	16(Ca)	2.54 ± 0.13	2.44 ± 0.10	1.68E-16

6	26(CK-MB)	9.77 ± 4.76	6.20 ± 3.25	3.56E-15

7	2(ALB)	52.19 ± 2.49	50.47 ± 2.42	1.54E-14

8	39(K)	4.05 ± 0.33	4.35 ± 0.36	1.63E-14

9	37(ADA)	11.5 ± 2.23	10.38 ± 2.09	4.28E-10

10	31(LP(a))	14.15 ± 10.66	11.63 ± 9.11	1.03E-09

11	19(CHOL)	4.12 ± 0.64	3.90 ± 0.69	1.11E-09

12	38(Na)	144.50 ± 2.74	142.86 ± 2.47	3.51E-09

13	28(APOC2)	2.56 ± 1.22	2.87 ± 1.42	2.13E-06

14	22(UIBC)	32.21 ± 9.89	36.50 ± 10.84	3.41E-06

15	15(LDL)	2.03 ± 0.51	1.91 ± 0.53	8.71E-06

16	34(FRUC)	169.57 ± 29.52	181.18 ± 23	9.90E-06

17	6(GLU)	3.62 ± 0.46	4.26 ± 1.97	4.90E-05

18	36(TBA)	169.57 ± 29.52	181.18 ± 23	8.03E-05

19	41(TIBC)	54.99 ± 8.68	56.99 ± 7.56	0.000485

20	1(TP)	79.82 ± 5.08	78.02 ± 5	0.000566

21	25(APOB)	0.85 ± 0.2	0.82 ± 0.22	0.000977

22	23(Fe)	22.79 ± 7.02	20.49 ± 6.94	0.001989

23	4(AST)	22.38 ± 5.07	21.14 ± 4.97	0.004223

24	33(PLIP)	2.43 ± 0.34	2.36 ± 0.36	0.006711

25	12(CK)	171.81 ± 117.62	142.78 ± 100.49	0.009752

26	27(APOA2)	27.56 ± 4.34	28.60 ± 4.38	0.021823

27	3(ALT)	18.45 ± 8.68	19.98 ± 10.68	0.023629

28	8(Cr)	79.44 ± 9.34	78.40 ± 8.23	0.035143

29	29(APOC3)	6.94 ± 2.07	7.47 ± 3.45	0.041523

We have compared the ranked lists of features by SVM-RFE and by paired t-test, and found that among the top nine features in the two lists, seven of them are common to both lists. The difference is mostly due to the way that the features are selected in the two methods, where SVM-RFE uses more global information and the paired t-test bases solely on individual features in dependent of others in their feature selections. We believe that the substantial smaller set of features that our method selected, compared to the other methods, provides a more focused and informative subset of features for further studies.

### Clinical Implication

Our analysis above identified a number of blood chemistry measures with consistent and substantial changes across all the seamen surveyed before and after their ocean voyages. The top 9 features selected by SVM-RFE are MAO, PHOS, CK-MB, Ca, LDH, FRUC, K, Na and ALB. Table 5 summarizes the changes in their mean values and standard deviation between before- and after voyage. Based on the findings, we noted the following:

**Table 5:** Comparison of blood chemistry measures before and after sailing

Measures(units)	**Before**()	**After**()
MAO(U/L)	0.85 ± 0.21	0.36 ± 0.18

PHOS(mmol/L)	1.17 ± 0.17	1.39 ± 0.16

CK-MB(U/L)	9.77 ± 4.76	6.20 ± 3.25

Ca(mmol/L)	2.54 ± 0.13	2.44 ± 0.10

FRUC(μmol/L)	169.57 ± 29.52	181.18 ± 23

LDH(U/L)	186.02 ± 37.99	158.20 ± 28

K(mmol/L)	4.05 ± 0.33	4.35 ± 0.36

Na(mmol/L)	144.49 ± 2.74	142.86 ± 2.47

ALB(g/L)	52.19 ± 2.49	50.47 ± 2.42

Creatine Kinase (CK) is typically present in the cytoplasm and mitochondria of organs such as heart, muscle and brain; and it is directly related to cellular energy conversion, muscle contraction and regeneration of ATP. It reversibly catalyzes the phosphoryl transfer reaction between creatine and ATP. We found that after the occean voyage, the mean value of CK reduced to 142.46 U/L from 171.68 U/L measured before sailing. This is probably due to the lack of physical exercise by the seamen, which ultimately led to the reduced demand for energy of the body muscle, as well as the reduction of CK. As one of its isoenzymes, the mean value of CKMB is reduced to 6.2 U/L from 9.73 U/L, the variation tendency is the same with CK. Lactate dehydrogenase (LDH) is a key enzyme in the glycolytic process. It exists in virtually all organs, particularly in liver, kidney, cardiac muscle, skeletal muscle, pancreas and lung. In anaerobic conditions, the regeneration of NAD+ is completed by the reaction in which LDH catalyzes pyruvic acid to become lactic acid; and LDH can also catalyze lactic acid to become pyruvic acid, with hydrogen being transferred to its coenzyme to become NADH. The mean value of LDH changed from 185.87 U/L to 158.15 U/L from pre-sailing to post-sailing, which could be due to the reduction of physical exercise intensity of seamen during the ocean voyage, having led to the reduction of energy supplied by the glycolytic process except for the normal aerobic metabolism of the body.

ALB (albumin) is made by the liver, and decreased serum albumin may indicate liver diseases as well as kidney disease, which allows albumin to escape into the urine.

Decreased ALB could also be explained by malnutrition or a low protein diet [21]. The altered ALB levels often suggest changed liver metabolism. Increased protein intake during ocean voyage may be needed in the seamen's diet.

Fructosamine (FRUC) is a substance formed from plasma protein during the life cycle of glucose. Since the half-life for plasma protein is 17 days, the measured FRUC level reflects the blood glucose level generated by the food taken in the 1-3 weeks prior to the voyage. Hence the observed change of FRUC from 169.74 μmol/L to 181.41 μmol/L after sailing probably reflects the type of food taken during the ocean voyage, which suggests that seamen should take less sugar during their future ocean voyage.

The observed change in the levels of inorganic ions, Ca, PHOS, K and Na may be caused by electromagnetism radiation and stress during the sailing. Though they cannot be used to diagnose specific diseases, their decreased levels generally indicate the poor state of the seamen's health. In the detected electrolyte, the level of serum sodium (Na) decreased obviously, possibly due to that Na ran off during sweating without being supplemented properly. In addition, calcium (Ca) is lacking in their diet, special measures should be adopted in their food preparation.

## Conclusions

The possible effects of long-distance ocean voyage on seamen's health are receiving increasingly more attention in recent years. Living on ship with confined environments, ocean-going seamen could suffer from various health problems due to abnormal electromagnetism radiation, great temperature changes, poor diet structure, which may cause subtle changes in physiological and psychological functions in their bodies. In this study, we have used an SVM-RFE approach to identify important blood chemistry measures with significant and consistent changes before and after a voyage. A number of features have been identified to have such changes, such as MAO, PHOS, CK-MB, Ca and FRUC. Their identification provides important clues about how ocean voyage may affect seamen's health. Our findings could provide useful guidance for making necessary changes in their living environments, food preparation and exercise routines.

## Competing interests

The authors declare that they have no competing interests.

## Authors' contributions

YML was responsible for the analysis and the draft of the manuscript. YHG collected the data and participated in the drafting of the part of 'clinical implication' of the manuscript. ZBC carried out the data analysis. JC conceived the study, participated in the design and coordination of the study and the revision of the manuscript. ZND participated in the collection of the data. YPT participated in the collection of data and gave medical analysis for the clinical implication. YX participated in the design and the revision of the manuscript. All authors read and approved the final manuscript.

## Pre-publication history

The pre-publication history for this paper can be accessed here:

http://www.biomedcentral.com/1472-6947/10/13/prepub
